# Calcium Intake and Nutritional Adequacy in Spanish Children: The ANIVA Study

**DOI:** 10.3390/nu9020170

**Published:** 2017-02-21

**Authors:** Nuria Rubio-López, Agustín Llopis-González, María Morales-Suárez-Varela

**Affiliations:** 1 Unit of Public Health, Hygiene and Environmental Health, Department of Preventive Medicine and Public Health, Food Science, Toxicology and Legal Medicine, University of Valencia, 46100 Valencia, Spain; nuria.rubio@uv.es (N.R.-L.); agustin.llopis@uv.es (A.L.-G.); 2 Biomedical Research Center Network on Epidemiology and Public Health (CIBERESP), 28029 Madrid, Spain

**Keywords:** children, nutritional intake, calcium, nutrients intake

## Abstract

Calcium is an important nutrient for child development. The main objective of this study was to assess calcium intake and its adequacy with dietary reference intake (DRI) in Spanish children. The ANIVA (Antropometría y Nutrición Infantil de Valencia) study is a descriptive cross-sectional study. During two academic years 2013–2014 and 2014–2015, 1176 schoolchildren aged 6–9 years were selected from 14 primary schools in Valencia (Spain). Three-day food records were used to assess dietary intake, completed by parents/guardian. Anthropometric data (weight and height) were evaluated in all subjects. Nutritional intake was compared to estimated average requirements (EARs) and adequate intake (AI) values to determine nutritional adequacy. A percentage of 25.77% had inadequate calcium intake, and a significantly higher prevalence was observed in girls (*p* = 0.006). Adequate calcium intake showed a positive association with the height *z*-score (*p* = 0.032). When assessing dietary patterns, schoolchildren with adequate calcium intakes had better nutritional adequacy in all nutrients, except cholesterol (*p* = 0.086) and fluorine (*p* = 0.503). These results suggest a public health problem that must be addressed through nutrition education programs to increase intake of calcium-rich food and to correct the associated dietary pattern.

## 1. Introduction

Calcium is one of the key minerals needed to ensure optimal bone health and teeth, and can be especially important during growth spurts [1]. Calcium plays a fundamental role in many organism functions, such as hormone secretion stimulation, participation in contracting muscles and nerve impulse transmissions, immune system, mental capacity, and learning [2,3]. However, its most important role is in bone skeletal development in childhood and adolescence. Therefore, ensuring adequate calcium intake can help minimize growth problems, prevent osteoporosis and osteopenia, and protect against fractures [4,5]. Calcium appears to have other beneficial functions for health. Some studies have linked calcium intake to the prevention of obesity, hypertension, kidney stones, insulin resistance, colon cancer, etc. [6,7].

The human body does not synthesize minerals and its presence depends exclusively on intake from diet. From the calcium content of foods, only 20%–40% of total calcium is absorbed [8]. Calcium bioavailability is favored with adequate intakes of lactose, vitamin D, fat, proteins, vitamin C, and an acid medium [1,8]. Conversely, its bioavailability lowers with foods rich in oxalic and phytic acids [1,8]. Therefore, an adequate calcium intake and other nutrients is essential for healthy growth. In childhood, one of the most important nutritional risks is poor eating habits, and three main agents intervene in their adoption: family, mass media, and school [9,10]. Poor eating habits include skipping meals, eating lots of processed food, and following fad diets [9]. One of the consequences of these poor eating habits is inadequate calcium intake, which is so fundamental in this growth stage.

Several studies performed in Spanish schoolchildren [6,11] have observed that calcium intake was generally below the dietary reference intake (DRI) [12,13]. Valencian Community representative data on calcium intake for children and the proportions that meet DRI are lacking.

Nutrition intake estimates can inform clinicians about key nutrient intakes during a critical growth period, and can also provide a basis for population level estimates that can support the efforts of prevention programs and health control.

Therefore, the main objective of the present study was to assess calcium intake and its adequacy for DRI in Valencian children.

## 2. Samples and Methods

### 2.1. Participants

ANIVA (Antropometría y Nutrición Infantil en Valencia; the Valencian Anthropometry and Child Nutrition), a descriptive cross-sectional study, was conducted in schoolchildren aged 6–9 years who went to one of the 14 participating primary schools in the province of Valencia. The sample size is higher than the cohort ANIVA study [14,15] because data collection took place over two academic years: 2013–2014 and 2014–2015. Children were selected by random cluster sampling in schools, and stratified by sex and school type (i.e., public vs. private). 

Before we started the study, it was orally presented to the Consejo Escolar (Board of Governors) of each participating school. Then, a letter was sent to the parents of all the children invited to participate in the study, which outlined the study goals and procedures and that data collected would be kept confidential in line with Spanish data protection regulations, and informed consent was thus obtained. 

For all children, the following inclusion criteria were applied: (a) the child had to be between 6 and 9 years old; (b) children who studied primary education at one of the 14 selected schools; (c) parents or legal guardians had to agree to the participation of the child and give written informed consent. The exclusion criteria were (a) a clinical diagnosis of chronic disease with dietary prescription; (b) absence from school on the days arranged to take body weight and height measurements; (c) an incomplete nutritional record.

The initial sample included 1432 children of both genders, of whom 11.38% did not want to participate (*n* = 163). The subjects who provided incomplete questionnaire (*n* = 61) or did not present anthropometric measurements (*n* = 32) were not included in the study. The participation rate was 82.12% and the resulting final sample comprised 1176 children. The study protocol complied with Declaration of Helsinki Guidelines and was approved by the Secretaría Autonómica de Educación, Conserjería de Educación, Cultura y Deporte of the Generalitat Valenciana, Valencia, Spain (2014/29630; the Regional Valencian Secretary of Education, Culture and Sport).

### 2.2. Examination Protocol and Measurements

Through a questionnaire completed by parents/guardian, we obtained information about child’s age, sex, medical history, medication, use of vitamin and mineral supplements, any special dietary restrictions, and food allergies. At the same time, parents/guardians were provided with details of how to assess the food and drinks consumed by their child. They were asked to record estimated portion sizes for each ingested item. The same training was provided to the caregivers responsible for children in school dining halls. This was essential to obtain reliable data. Parents were asked to submit food labels with ingredients, brands, added ingredients, and recipes of homemade dishes whenever possible. In addition, for information and support, they were given an email and telephone number, which they could write or call to help resolve any issues that arose while completing the diary.

### 2.3. Dietary Assessment

On a form provided, parents and guardians were asked to record all the foods and drinks consumed by their child over a three-day period (Supplementary Materials), including one non-working day (e.g., Sunday or Saturday) [16,17,18]. In order to calculate intake of energy and macro- and micronutrients, researchers entered data from these food records into a software, DIAL^®^ (DIAL^®^, v2.16, Madrid, Spain) [19]. This software was developed by the Department of Nutrition and Dietetics at the Madrid Complutense University (Spain), which has been previously validated in Spain to assess diets and to manage nutritional data. This open software includes a list of some enriched/fortified foods commonly available in Spain to which other foods can be added to the database. With this feature, we were able to include the nutritional composition of packaged foods taken from food labels that parents and guardians submitted.

### 2.4. Estimate of Nutrients Adequacy/Deficiency

DRIs [17,20,21] are used to assess and plan individual and group nutrient intakes of healthy people and include the following reference values: the estimated average requirement (EAR), the recommended dietary allowance (RDA), the adequate intake (AI), and the tolerable upper intake level (UL), as well as Estimated Energy Requirements (EERs) for energy and Acceptable Macronutrient Distribution Ranges (AMDRs) for macronutrients. For each nutrient, children were categorized as being at risk of inadequate intake based on whether or not they met the corresponding nutritional targets [12] and DRIs [13] proposed for the Spanish population. In our study, individual intake was compared to the EAR, which is the average amount of nutrient intake estimated to meet the requirements of one-half of the healthy individuals in a particular life stage and sex group. Using the RDA (requirements for 97%–98% of healthy individuals) was considered but discarded given that it would set a very strict standard and would lead to an overestimation of the number of children with inadequate calcium intake. 

The probability of adequate intake for each micronutrient [17] and [12,13] was calculated from a *z*-score as follows: *z*-score = (estimated nutrient intake − EAR)/SD, where SD indicates EAR standard deviation [21].

Specifically, we used AI values for nutrients for which EARs have not been available (fiber, fluoride, manganese, potassium, and pantothenic acid). The percentage of energy provided by proteins, lipids and carbohydrate was also calculated and compared with AMDRs.

For nutrients presumed to be detrimental with high intakes (e.g., lipids, cholesterol), the opposite interpretation was applied, which means the diet was considered inadequate if the limit was exceeded and adequate if the intake was below the limit. 

### 2.5. Anthropometric Measurements

During school hours, weight (in kilograms) and height (in centimeters) were recorded of each of the child using a digital electronic scale (OMRON BF511^®^, Tokyo, Japan; precision: 0.05 kg) and a stadiometer (Seca 213^®^, Hamburg, Germany; precision: 1 mm), respectively. For both measurements, participants stood barefoot and wore minimal clothes, which agrees with the norms set up by the World Health Organization [22]. With these data, we calculated BMI-for-age (*z*-score), weight-for-age (*z*-score), and height-for-age (*z*-score) with the WHO Anthro software, v.3.2 (Geneva, Switzerland) [23]. Based on the obtained percentile ranking, BMI was used to classify children into one of four categories [24]: underweight (≤5th percentile), normoweight (>5th to <85th percentiles), overweight (≥85th to <95th percentiles), or obese (≥95th percentile).

### 2.6. Statistical Analysis

Continuous variables were expressed as the means (standard deviations, SD), whereas categorical variables were expressed as frequency (percentages, %). The Kolmogorov–Smirnov test was used to determine the normality of the distribution of the examined variables. For the comparison of the means between groups, a one-way analysis of variance was used with the Bonferroni rule to correct for inflation in the type 1 error due to multiple post hoc comparisons. The chi-square test was run to explore the association between categorical variables, and the two-sample z-test for proportions for multiple post hoc comparisons. All the *p*-values were two-tailed, and statistical significance was set at the conventional cut-off of *p* < 0.05. Data were inputted into an Excel spreadsheet using the double-data entry to minimize the risk of errors and were then transferred to the IBM SPSS software, version 17.0 (SPSS Inc., Chicago, IL, USA).

## 3. Results

Table 1 reports the schoolchildren’ baseline characteristics according to calcium intake. The sample included 1176 schoolchildren made up of 561 boys (47.7%) and 615 girls (52.3%). Of all schoolchildren studied, 25.8% presented with inadequate calcium intake. The prevalence of inadequate calcium intake was significantly higher for girls (*p* = 0.006). We found a statistically significant difference only in height (*p* = 0.001) and height *z*-score (*p* = 0.032); children with adequate calcium intake were significantly taller compared to children with inadequate calcium intake. Although the differences were not significant, the results suggested that children with inadequate calcium intake also had higher BMI *z*-score compared to children with adequate calcium intake.

Table 2 summarizes the proportions of nutritional inadequacy according to calcium intake and gender. The results of the present study showed that mean calcium intake of children with adequate calcium intake was 1081.10 ± 232.79 mg/day; in contrast, that of children with inadequate calcium intake was 649.44 ± 118.11 mg/day. 

Comparing the groups of girls and boys with adequate calcium intake, in general, girls showed worse nutritional adequacy; however, only significant differences for total energy (*p* = 0.003) and zinc (*p* = 0.042) were observed. However, when we compared the groups of girls and boys with inadequate calcium intake, significant differences were observed for carbohydrates (*p* = 0.002), proteins (*p* = 0.033), lipids (*p* = 0.024), cholesterol (*p* = 0.021) and vitamin A (*p* = 0.022). In this case, girls also had worse nutritional adequacy than boys. Therefore, better nutritional adequacy was identified in boys, independently of calcium intake.

In general, regarding the comparison of nutritional inadequacy (regardless of gender) between children with adequate calcium intake and inadequate calcium intake, we observed that children with adequate calcium intake had significantly lower prevalence of inadequacy for total energy, carbohydrates, protein, lipids, fiber, thiamin, vitamin B_6_, biotin, folic acid, vitamins C, A, D, and E, magnesium, iron, zinc, and iodine (*p* < 0.05) than children with inadequate calcium intake. 

## 4. Discussion

Adequate calcium intake in childhood is key for optimal growth and development. Our findings indicate that one in four children aged 6–9 years is not consuming enough calcium to meet Spanish recommendations. Prevalence of calcium intake lower than the recommendations was significantly higher in girls. In line with other Spanish studies [6,11] conducted in recent decades, which also considered this geographical area, we observed that this problem remains. Suarez-Cortina et al. [11] in a representative sample of 1176 Spanish children (5–12 years), identified inadequate calcium intake in 15.3% of the sample. In contrast, Ortega et al. [6] indicated a higher prevalence of low calcium intake, and 76.7% of the sample (7–11 years) had inadequate calcium intake. Both studies [6,11] reported that inadequate calcium intake was more frequent in girls than boys, which is similar to our results. Other studies in Europe, such as that performed by Moreira et al. [25] in Portuguese children or the study carried out by Merkiel [26] in Polish children, reported a high prevalence of inadequate calcium intake.

Regarding anthropometric assessments, the schoolchildren with adequate calcium intake were taller than children with inadequate calcium intake. Although there are other variables that influence the height of an individual, such as genetics, Bhargava [27], and Cao et al. [28] have described that calcium intake has been associated with higher bone mineral densities and content, and with taller individuals. Although the differences were not significant, the results showed an inverse relationship between calcium intake and the BMI *z*-score, and children with inadequate calcium intake had higher BMI *z*-scores. 

An inadequate calcium intake at this age has clinical significance in relation to anthropometric development. Inadequate calcium intake can at times be due to low food intake. However, in our study, the children with inadequate calcium intake are shorter but have higher BMI *z*-scores, which shows that the total amount of food intake is not the problem. As previously mentioned, these *z*-scores are age-adjusted, and the stage of growth and maturation of the children is therefore accounted for. These children present an inadequate calcium intake due to a poor dietary pattern. The continuous adherence to a poor dietary pattern, which is especially key for growth and development at this age, could result in future health complications related to bone and organ development. 

Based on the Spanish Dietary Recommendations [12,13] for schoolchildren aged 6–9 years, children with adequate calcium intake, especially boys, had significantly lower prevalence of inadequacy for total energy, carbohydrates, protein, lipids, fiber, thiamin, vitamin B_6_, biotin, folic acid, vitamins C, A, D, and E, magnesium, iron, zinc, and iodine compared to children with inadequate calcium intake. We identified that children with adequate calcium intake have higher nutritional adequacy. Campmans-Kuijpers et al. [29] similarly observed that children who had a high intake of one nutrient tended to have adequate intakes in the other nutrients. This possibly occurs due to the fact that eating patterns with high calcium content normally also have high vitamin and mineral contents. Therefore, an inadequate calcium intake could be an indicator of an inadequate intake of other macro and micronutrients. 

Overall, in both the adequate and inadequate calcium intake groups, we found a high percentage of nutritional dietary excess for proteins. Both groups presented protein intakes noticeably above the EAR, which for proteins is 15% TEV. When the dietary pattern contains high protein intake, particularly from omnivorous sources, urinary calcium excretion increases and, if maintained, will result in sustained hypercalciuria [30,31]. The most important regulator of urinary calcium is dietary protein [32,33]. To date, the majority of calcium balance studies in humans have not detected an effect of dietary protein on intestinal calcium absorption or serum parathyroid hormone [28,34]. However, protein intakes below the DRI can be detrimental for bone formation and bone conservation throughout adulthood [30]. 

Likewise, the fat intake for all our schoolchildren was higher than that recommended given the change in dietary patterns that is currently occurring [35]. Carbohydrate and fiber intakes were below those recommended in more than 90% of the studied sample, results that are similar to those reported in the studies of Khan et al. [36] and Storey [37]. Therefore, an unbalanced dietary pattern was observed for the studied population, as it presented a pattern with a high intake of energy, proteins, and fats and a low intake of carbohydrate and fiber. Regarding micronutrients, poorer nutritional adequacy was noted among the schoolchildren with inadequate calcium intake, similarly described by Campmans-Kuipers et al. [29].

According to the literature, calcium uptake is affected by the intake or bioavailability of other nutrients, such as phosphorus and protein [6,38]. Thus, poor calcium absorption can be magnified if the calcium/protein and calcium/phosphorous ratios are inadequate [38]. Specifically, more than 99% of the studied children had a calcium/phosphorus ratio below 1 and a calcium/protein ratio below 20, which are in line with Ortega et al. [6]. These results once again reveal a low calcium intake associated with a high intake of phosphorus and proteins, which may have an adverse effect on calcium utilization and bone mass maintenance. When ratios are below recommendations, there is less calcium bioavailability to form insoluble salts, which can be unfavorable for bone formation and development in children [38].

Vitamin D is a hormone involved in the regulation of calcium homeostasis, as it regulates calcium absorption from the gastrointestinal system. Low vitamin D levels in the body make calcium absorption difficult [39]. However, vitamin D can be synthesized in our body from a derivate of cholesterol as a result of exposure to solar ultraviolet-B irradiation [34]. In this study, vitamin D deficiency is reduced given that even in winter the Mediterranean region has high levels of sunshine [40]. 

Special attention should be paid to the observed nutrient deficiencies, and their intake should be actively reinforced by primary care and schools since such nutrients act directly on child growth and development. Intake of certain foods can be improved by setting up Food Education Programs to encourage healthy eating habits such as higher daily intakes of fish, fruit, and vegetables, and eating a decent breakfast [14]. The school environment, along with family and community environments, are the most influential educational areas, where healthy eating and lifestyle habits are acquired. Attitudes to be taken by schools in terms of nutritional aspects should be intrinsically exemplified to satisfy their educational purpose, and to consequently help avoid nutritional deficiencies in children.

### Study Limitations

This study is not without its limitations. As it is a cross-sectional study, it is difficult to assert a possible cause–effect relation between calcium intake and its effect on anthropometric measurements and nutritional adequacy as measurements were taken at the same time. Future large-scale longitudinal studies on this subject are needed to examine the causal relation between nutritional deficiency and anthropometric measurements. Our study presents strong internal validity given its high participation rate. The information obtained from the food records used for nutritional assessment purposes was of good quality. Parents and schools were very interested in the study and were provided with information so they could complete food records. We understand that these factors compensate the limitations in our generalizability and external validity.

## 5. Conclusions

Calcium intake was inadequate in 25.8% of the studied sample, which can affect growth during childhood. Height was related inversely with calcium intake. Deficiencies in the intake of a major micronutrient, such as calcium, is likely to reflect inadequate intakes of other macro and micronutrients. It is important to pay attention to these nutritional deficiencies, as each nutrient plays important roles in the human body. Inadequate mineral intakes affect growth and physical development, the body’s immune system, mental capacity, and learning. An increase in the consumption of dairy products, cereals, vegetables, and food items fortified with calcium seems necessary to achieve adequate calcium intake. Educational nutrition programs are necessary to encourage healthy eating habits. Future studies about this theme are necessary to more specifically identify calcium deficiencies in the diet of today’s schoolchildren and their effect on future adults.

## Figures and Tables

**Table 1:** Schoolchildren’s anthropometric characteristics according to calcium intake.

	Adequate Calcium Intake (*n* = 873, 74.2%)		Inadequate Calcium Intake (*n* = 303, 25.8%)		*p*-Value
Variables	M/*n*	SD/%	M/*n*	SD/%	
Gender *					
Boys	437	50.1	124	40.9	0.006
Girls	436	49.9	179	59.1	0.006
Age (years)	7.38	1.06	7.44	1.11	0.401
Weight (kg)	30.42	7.51	30.32	7.68	0.843
Height (m)	1.31	0.09	1.29	0.09	0.001
BMI *					
Underweight	53	6.1	16	5.3	0.614
Normoweight	472	54.1	173	57.1	0.361
Overweight	181	20.7	59	19.5	0.639
Obesity	167	19.1	55	18.1	0.708
Weight *z*-score	1.11	1.24	1.07	1.31	0.634
Height *z*-score	0.90	1.34	0.71	1.29	0.032
BMI *z*-score	0.88	2.24	0.90	1.33	0.883

M: Mean; *n*: number; SD: Standard Deviation; %: percentage; BMI: Body Mass Index. * Percentage was done in columns. Means were compared with the use of a ANOVA test, and proportions with the use of a chi-square test. *p*-value < 0.05: considered statistically significant.

**Table 2:** Nutritional inadequacy according to calcium intake and gender.

Nutrients	DRIs	Adequate Calcium Intake (*n* = 873; 74.2%)			Inadequate Calcium Intake (*n* = 303; 25.8%)			*p*-Value (Total)
		Boys (*n* = 437)	Girls (*n* = 436)	*p*-Value (Boy vs. Girl)	Boys (*n* = 124)	Girls (*n* = 179)	*p*-Value (Boy vs. Girl)	
		*n* (%)	*n* (%)		*n* (%)	*n* (%)		
Total energy	<EER	49 (13.2)	81 (21.5)	0.003	54 (47.0)	84 (51.5)	0.452	0.001
Carbohydrates	<EAR	358 (96.8)	365 (97.1)	0.802	102 (88.7)	159 (97.5)	0.002	0.001
Protein ^a^	>EAR	268 (72.4)	282 (75.0)	0.426	84 (73.0)	99 (60.7)	0.033	0.008
Calcium/protein ratio	<EAR	369 (99.7)	115 (100)	-	115 (100)	163 (100)	-	-
Fats ^a^	>EAR	328 (88.6)	331(88.0)	0.793	91 (79.1)	145 (89.0)	0.024	0.043
Cholesterol ^a,b^	>EAR	75 (20.3)	77 (20.5)	0.944	12 (10.4)	34 (20.9)	0.021	0.086
Fiber	<AI	342 (92.4)	360 (95.7)	0.055	112 (97.4)	160 (98.2)	0.987	0.014
Thiamin	<EAR	6 (1.6)	12 (3.2)	0.162	12 (10.4)	11 (6.7)	0.272	0.001
Riboflavin	<EAR	0 (0.0)	2 (0.5)	-	24 (20.9)	35 (21.5)	0.904	-
Niacin	<EAR	0 (0.0)	0 (0.0)	-	1 (0.9)	0 (0)	-	-
Pantothenic acid	<AI	0 (0.0)	1 (0.3)	-	1 (0.9)	3 (1.8)	0.874	-
Vitamin B_6_	<EAR	1 (0.3)	2 (0.5)	0.989	8 (7.0)	6 (3.7)	0.219	0.001
Biotin	<EAR	1 (0.4)	2 (0.7)	0.628	5 (6.8)	6 (5.4)	0.622	0.001
Vitamin B_12_	<EAR	0 (0.0)	1 (0.3)	-	0 (0.0)	1 (0.6)	-	-
Folic acid	<EAR	100 (27.0)	103 (27.4)	0.910	52 (45.2)	84 (51.5)	0.299	0.001
Vitamin C	<EAR	23 (6.2)	28 (7.4)	0.505	14 (12.2)	22 (13.5)	0.746	0.017
Vitamin A	<EAR	10 (4.1)	19 (7.1)	0.064	10 (13.7)	28 (25.0)	0.022	0.001
Vitamin D	<EAR	298 (80.5)	287 (76.3)	0.162	103 (89.6)	151 (92.6)	0.369	0.001
Vitamin E	<EAR	132 (35.7)	148 (39.4)	0.299	64 (55.7)	84 (51.5)	0.498	0.001
Phosphorus	<EAR	0 (0.0)	2 (0.5)	-	0 (0.0)	0 (0.0)	-	-
Calcium/phosphorus ratio	<EAR	368 (99.5)	373 (99.2)	0.986	115 (100)	163 (100)	-	-
Magnesium	<AI	1 (0.3)	3 (0.8)	0.627	13 (11.3)	14 (8.6)	0.451	0.001
Iron	<EAR	21 (5.7)	34 (9.0)	0.078	27 (23.5)	43 (26.4)	0.583	0.001
Zinc	<EAR	7 (1.9)	17 (4.5)	0.042	24 (20.9)	45 (27.6)	0.200	0.001
Iodine	<EAR	253 (68.4)	276 (73.4)	0.131	111 (96.5)	156 (95.7)	0.975	0.001
Selenium	<EAR	0 (0.0)	0 (0.0)	-	0 (0.0)	0 (0.0)	-	-
Fluoride	<AI	356 (96.2)	363 (96.5)	0.811	111 (96.5)	153 (93.9)	0.878	0.503

*n*: number; TEV: Total Energy Value; EAR: estimated average requirement; AI: adequate intake. DRIs: EAR: carbohydrates (50%–60% TEV), protein (10%–15% TEV), calcium/protein ratio (20 mg/g), fats (30%–35% TEV), thiamin (0.8 mg/day), riboflavin (1.2 mg/day), niacin (12 mg/day), vitamin B_6_ (1.4 mg/day), biotin (12 µg/day), vitamin B12 (1.5 µg/day), folic acid (200 µg/day), vitamin C (55 mg/day), vitamin A (400 µg/day), vitamin D (5 µg/day), vitamin E (8 mg/day), calcium (800 mg/day), phosphorus (700 mg/day), calcium/phosphorus ratio (1 mg/mg), iron (9 mg/day), zinc (10 mg/day), iodine (90 µg/day), selenium (30 µg/day); AI: fiber (25 mg/day), pantothenic acid (3 mg/day), magnesium (180 mg/day), fluoride (1000 µg/day). *p* < 0.05 was considered statistically significant (Student’s t-test). It was considered inadequate when carbohydrate intake <50% TEV, protein >15% TEV, and fats >35% TEV. ^a^ Intakes failed to meet recommendations if they were under DRIs, except for protein, fats, and cholesterol, for which inadequate intakes were those over DRIs or nutritional targets for Spanish people, respectively. ^b^ The DRI for cholesterol was not determinable. Instead the Spanish population target of 100 mg/1000 kcal was considered. *p* < 0.05 was considered statistically significant.

## Supplementary Materials

The following are available online at http://www.mdpi.com/2072-6643/9/2/170/s1.

## Abbreviations

The following abbreviations are used in this manuscript:

AI	adequate intake
AMDR	acceptable macronutrient distribution ranges
ANIVA	Antropometría y Nutrición Infantil de Valencia
BMI	body mass index
CI	confidence interval
DRI	dietary reference intake
EAR	estimated average requirement
EER	estimated energy requirement
M	mean
*n*	number
RDA	recommended dietary allowance
SD	standard deviation
UL	tolerable upper intake levels
