# Supplementary material for: Calcium Intake and Nutritional Adequacy in Spanish Children: The ANIVA Study
Source: Nutrients. 2017 Feb 21;9(2):170. doi: 10.3390/nu9020170 (PMC5331601; doi:10.3390/nu9020170)
Supplement: Supplementary file 1 [file nutrients-09-00170-s001.docx]

ANIVA Study: THE FOOD INTAKE RECORD QUESTIONNAIRE

| **PERSONAL DATA OF THE CHIL** | | |
| --- | --- | --- |
| **NAME AND SURNAME:** | | |
| **SCHOOL:** | | **COURSE:** |
| **DATE OF BIRTH:** | **POSTAL CODE RESIDENCE:** | |

INSTRUCTIONS:

In this questionnaire, please make a note of all the food items, drinks, dietary supplements and prepared food you eat over a 3-day period, one of which must be Saturday or Sunday. There are two sheets for each day: the first one is to write down all the food items eaten in the morning; the second one is to make a note of everything you eat in the afternoon/evening. All food items, drinks and ready meals must be recorded, without forgetting those you may have eaten in between meals: snacks, portions, solutions, sweets, etc. Do not forget water or other drinks you have drunk with a meal or in between meals.

In the first column of each sheet, you should make a note of the plate or the overall menu. Please also indicate the way items were cooked (fried potatoes, grilled fillet, etc.).

In the second column, provide details of all the ingredients in each meal eaten on one day, and provide as many details as possible about the food items eaten:

- Indicate the brand name if you know it.
- Specify if each food item is normal, low-calorie or enriched. For example, if milk was full-cream, skimmed or semi-skimmed, if yoghurt was full-cream, skimmed or enriched, etc.
- Type of oil (olive, sunflower, etc.).
- Butter or margarine.
- White bread, brown bread, sliced bread.

Please indicate the amount of each food item you have eaten as accurately as possible. The best results are obtained by indicating the weight of each food item eaten, if it was weighed raw or cooked, and do not forget to discount or make a note of any leftovers. If weighing food is possible, please specify the measures; e.g., cups drunk, glasses drunk, spoonfuls, etc:

- Drinks: quantities can be expressed in cups, glasses, shorts, etc., if you are unaware of the volume measures.
- Soups, broths or purées: use cups or platefuls (large, medium or small).
- Meat, fish, fruit, and vegetables: estimate the amount eaten by taking into account the quantity purchased and the number of items or portions purchases included. If this is not possible, then please indicate the number and size of the portions eaten.
- Pulses: consider the size of the container and then divide it among the number of resulting rations if they were all equal. Otherwise, indicate the approximate ration size by making a note of the number of spoonfuls or bowls served, or the plate/dish size.
- Oil: indicate the number and type of spoons used (soup, dessert or coffee) added to meals. If food was fried, deduct the number of spoonfuls left in the frying pan from those poured into the frying pan, and distribute the resulting amount among the number of fried food items or fellow diners if everyone ate a similar amount.
- Sauces or sugar: For sugars, write down the number of spoonfuls, spoon size, and if level or heaped. For sauces, specify if completely eaten or if some was left on the plate.
- Bread: Indicate the number of slices, or pieces and their approximate size of bread eaten.
- Cooked meats: make a note of the number of slices and their thickness.
- For pre-cooked meals, please indicate the brand name and attach information on their composition, if available.
- For ready meals, supplements or dietary products, indicate the number of portion, packets, spoonfuls and brand name. If possible, attach a photocopy of its/their composition.

If in doubt or you need any explanation, please make a note of it on the rear side of the questionnaire sheets.

| **SUPPLEMENT FOOD OR VITAMIN THAT CONSUM YOUR CHILD** | | |
| --- | --- | --- |
| Put an X in the real option. If you select YES, please answer the questions. | | |
| **NO** |  |  |
| **YES** |  | **Supplement brand:** |
|  |  | **Consumption period:** |
|  | | **Amount/day:** |
|  |  | **Reasons intake:** |

| **DAY 1. DURING THE WEEK** | | | **DATE:** |
| --- | --- | --- | --- |
| **Menu** | **Details of ingredients and portion size or quantify (household measures or grams)** | | |
| **BREAKFAST** | | | |
| Example : chocolate milk and cookies + orange | | A glass of semi-skimmed milk enriched with calcium large ( 250ml ) with a dessert spoon of cocoa powder and 4 graham crackers + 1 large orange (pice, no juice) | |
| Place: | |  | |
| **BREAK** | | | |
| Example : Tomato and prosciutto sandwich + apple juice | Piece of bread (50g) with ½ sliced ​​tomato, 2 half slices of ham and drizzle olive oil (5-10ml) + brick 200ml of apple juice. | | |
| Place: |  | | |
| **LUNCH** | | | |
| Example: Lentils + breast with mixed vegetables + yogurt + bread. | | **1º**: half a bowl of lentils with 1 carrot , ½ potato, ½ sausage  **2º:** 2 small breast fillets with mixed vegetables (pea, carrot ... 100g). Sweetened yogurt and a slice of bread (25g). | |
| Place: | |  | |

| **DÍA 1. ENTRE SEMANA / LABORABLE** | | |
| --- | --- | --- |
| **Food (menu ingredients)** | | **Amount (g) or portion sizes** |
| **BREAK** | | |
| Example : mandarins + toast with jam | | 2 small mandarins + 4 fingers toasted white bread oven with a tablespoon of strawberry jam. |
| Place: | |  |
| **DINNER** | | |
| Example: grilled hake with artichoke and mashed potatoes. Note: we didn’t want dessert. | | Hake fillet ( approx . 125g ) grilled without oil with 3 artichoke hearts sauteed in oil 10ml per person. 50g flaked mashed potatoes (reconstituted in water) with a teaspoon of butter dessert. |
| Place: | |  |
| **FOOD BETWEEN MEALS** | | |
| Example 1: glass of white milk before bedtime.  Example 2: peanuts between lunch and dinner. | | Regular cup (200ml) of skimmed cow milk enriched with calcium.  Handful of peanuts (20g) fried with salt. |
| Place: | |  |
| **DAY 2. DURING THE WEEK** | | **DATE:** |
| **Menu** | **Details of ingredients and portion size or quantify (household measures or grams)** | |
| **BREAKFAST** | | |
| Example : chocolate milk and cookies + orange | | A glass of semi-skimmed milk enriched with calcium large ( 250ml ) with a dessert spoon of cocoa powder and 4 graham crackers + 1 large orange (pice, no juice) |
| Place: | |  |
| **BREAK** | | |
| Example : Tomato and prosciutto sandwich + apple juice | Piece of bread (50g) with ½ sliced ​​tomato, 2 half slices of ham and drizzle olive oil (5-10ml) + brick 200ml of apple juice. | |
| Place: |  | |
| **LUNCH** | | |
| Example: Lentils + breast with mixed vegetables + yogurt + bread. | | **1º**: half a bowl of lentils with 1 carrot , ½ potato, ½ sausage  **2º:** 2 small breast fillets with mixed vegetables (pea, carrot ... 100g). Sweetened yogurt and a slice of bread (25g). |
| Place: | |  |

| **DAY 2. DURING THE WEEK** | | |
| --- | --- | --- |
| **Food (menu ingredients)** | | **Amount (g) or portion sizes** |
| **BREAK** | | |
| Example : mandarins + toast with jam | | 2 small mandarins + 4 fingers toasted white bread oven with a tablespoon of strawberry jam. |
| Place: | |  |
| **DINNER** | | |
| Example: grilled hake with artichoke and mashed potatoes. Note: we didn’t want dessert. | | Hake fillet ( approx . 125g ) grilled without oil with 3 artichoke hearts sauteed in oil 10ml per person. 50g flaked mashed potatoes (reconstituted in water) with a teaspoon of butter dessert. |
| Place: | |  |
| **FOOD BETWEEN MEALS** | | |
| Example 1: glass of white milk before bedtime.  Example 2: peanuts between lunch and dinner. | | Regular cup (200ml) of skimmed cow milk enriched with calcium.  Handful of peanuts (20g) fried with salt. |
| Place: | |  |
| **DAY 3. WEEKEND** | | **DATE:** |
| **Menu** | **Details of ingredients and portion size or quantify (household measures or grams)** | |
| **BREAKFAST** | | |
| Example : chocolate milk and cookies + orange | | A glass of semi-skimmed milk enriched with calcium large ( 250ml ) with a dessert spoon of cocoa powder and 4 graham crackers + 1 large orange (pice, no juice) |
| Place: | |  |
| **BREAK** | | |
| Example : Tomato and prosciutto sandwich + apple juice | Piece of bread (50g) with ½ sliced ​​tomato, 2 half slices of ham and drizzle olive oil (5-10ml) + brick 200ml of apple juice. | |
| Place: |  | |
| **LUNCH** | | |
| Example: Lentils + breast with mixed vegetables + yogurt + bread. | | **1º**: half a bowl of lentils with 1 carrot , ½ potato, ½ sausage  **2º:** 2 small breast fillets with mixed vegetables (pea, carrot ... 100g). Sweetened yogurt and a slice of bread (25g). |
| Place: | |  |

| **DÍA 3. FIN DE SEMANA / FESTIVO** | |
| --- | --- |
| **Food (menu ingredients)** | **Amount (g) or portion sizes** |
| **BREAK** | |
| Example : mandarins + toast with jam | 2 small mandarins + 4 fingers toasted white bread oven with a tablespoon of strawberry jam. |
| Place: |  |
| **DINNER** | |
| Example: grilled hake with artichoke and mashed potatoes. Note: we didn’t want dessert. | Hake fillet ( approx . 125g ) grilled without oil with 3 artichoke hearts sauteed in oil 10ml per person. 50g flaked mashed potatoes (reconstituted in water) with a teaspoon of butter dessert. |
| Place: |  |
| **FOOD BETWEEN MEALS** | |
| Example 1: glass of white milk before bedtime.  Example 2: peanuts between lunch and dinner. | Regular cup (200ml) of skimmed cow milk enriched with calcium.  Handful of peanuts (20g) fried with salt. |
| Place:: |  |

- Ortega, R.M.; Requejo, A.M.; López-Sobaler, A.M. Modelos de cuestionarios para realización de estudios dietéticos en la valoración del estado nutricional. In Nutriguía Manual de Nutrición Clínica en Atención Primaria. Ortega RM and Requejo AM, editors; Ediciones Complutense, Spain, 2006; 456-459.
